# Supplementary material for: The Extracellular Matrix Regulates Invasion in Fusion-Negative Rhabdomyosarcoma via YAP–PIEZO1 Signaling Axis
Source: Cancers (Basel). 2026 Mar 4;18(5):827. doi: 10.3390/cancers18050827 (PMC12984940; doi:10.3390/cancers18050827)
Supplement: Supplementary file 1 [file cancers-18-00827-s001.zip › cancers-4100109-supplementary.pdf]

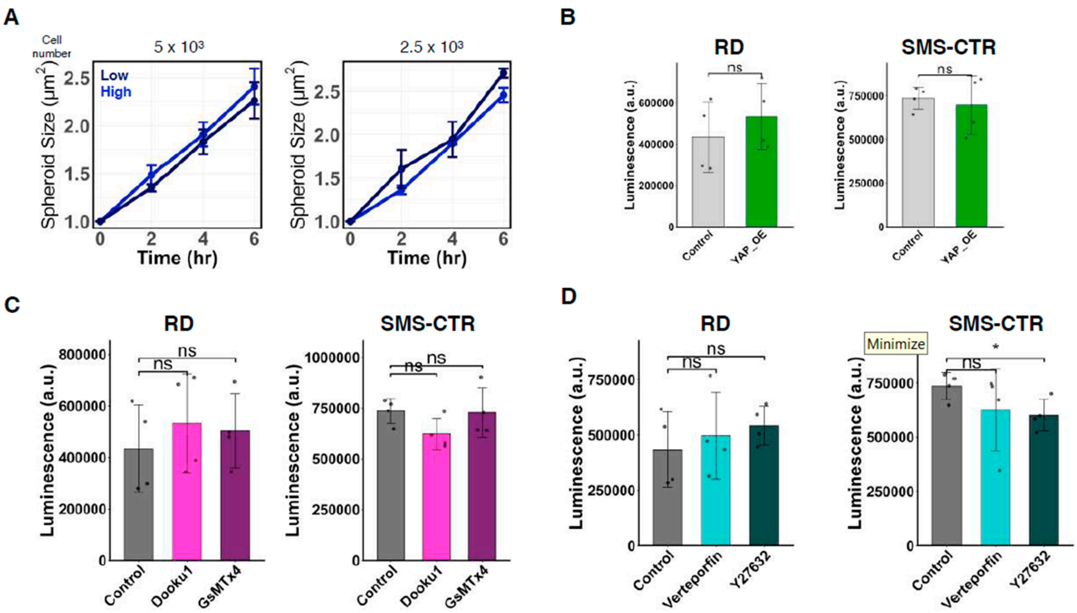

Supplementary Figure S1. Spheroid outgrowth independent of cell proliferation.
A. Quantitative analysis of FPRMS cell RH41 spheroid invasion over 6 hours. Error bars represent SEM from 5-6 independent spheroids. Statistical significance comparing low- and high-density groups at each time point was assessed using two-sided Student's t-tests and all condition displayed no statistical significance. **B-D.** Quantification of cell proliferation after 6 hours of treatment on high-density matrices. (B) Control and YAP-overexpression, (C) Control and calcium channel PIEZO1 inhibitors, and (D) Control and YAP inhibitors.

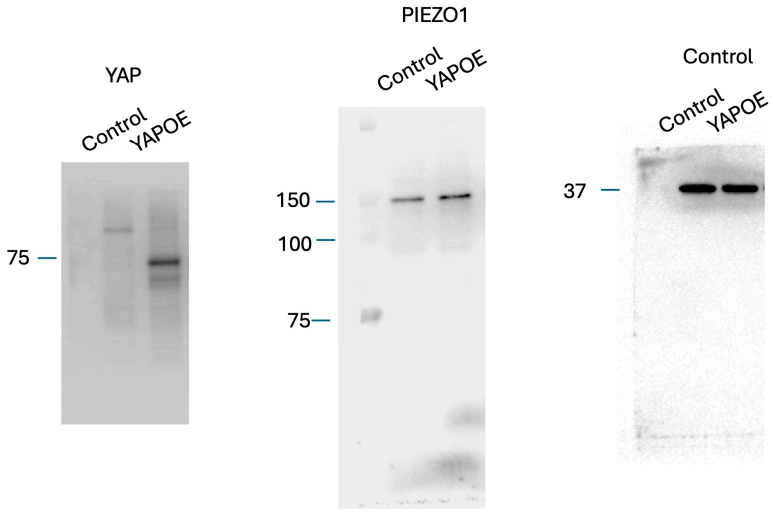

Supplementary Figure S2. Uncropped Western Blot images.
